# Proceedings of the Tennessee Dental Society

**Published:** 1879-03

**Authors:** H. E. Beach


### PROCEEDINGS OF THE TENNESSEE DENTAL SOCIETY.

*Held at Nashville, June 26th, 1878.*

*Continued from page 89.*

MORNING SESSION.

Dr. Noel's address being the order of business for this hour, the following discussion ensued:

Dr. W. J. Wilson, D. D. S., and J. U. Lee were elected to membership.

Dr. Freeman—I want to call the attention of the association to that part of the address that refers to dental legislation. The enactments of the legislature of other states is forcing upon us dental tramps, who are mere empirics, calling themselves doctors, who peddle their wares in connection with sewing machine needles and plaster toys. The interest of our patrons and the public demand that something shall be done. Many men, all over the country, who have been a failure at everything else, happen in a dental office, see a tooth extracted, for which a dentist receives a dollar, and straightway he gets a few old discarded instruments, and immediately starts out to practice dentistry, styling himself Doctor. Such men boast that they practice for the ignorant, and they are learning.

Letters are received here asking if they can be made dentists in one course of lectures.

Dr. Russell said he took the position of professor of oral surgery with great misgivings, but experience had proven it to be a step in the right direction.

It is the purpose of the college with which I am connected, to establish a dental department, which will fully qualify men to practice dentistry in all its departments.

Dr. Freeman suggested that the President's address be read. Which was done.

Dr. Cobb—The subject of dental legislation is one of great interest, and I have had much to do with it. Knowing that to practice dentistry intelligently men must feel this necessity in order to bring them up to the mark, and I favor the move to secure dental legislation by proper effort, not to keep men out of the profession, but to make them competent dentists. Think it is well to appoint a committee to bring the matter to an issue.

Dr. Russell—I see the necessity for dental legislation, but do not propose to discuss this matter. Neither do I come here to advocate the indorsement of it by this association. These things ought to be left to regulate themselves.

Dr. Sandusky thinks there never has been a time when dentistry was more prostituted than now. I think it a matter of great importance. I know a number of cases that have came under my notice of persons who are riding around practicing, who never had an hour's instruction, and what I say of dentistry will refer to a great extent to medicine.

Dr. Prewett—As I am from Kentucky my interest in the laws of Tennessee does not affect me, but I feel a deep interest in this direction. Although I came in the profession the old way, I have labored long and faithful to make myself proficient, and approve coming in the regular way. I think all students should receive a diploma, and if there is a dental school in Nashville I expect to attend and graduate.

Dr. Morgan—It is generally known that I am and have been an advocate of a high state of dental education, have long favored and advocated the teaching of dentistry in connection with general medicine. The statistics of last year shows that out of two thousand men who entered the dental profession only about two hundred were regular graduates. We are to a great extent responsible for this condition of things, and if there is ever a better condition it must be inaugurated by the members of this body.

Would be glad to see a dental department established in the Vanderbilt University, but the University will not now incur the expense requisite for it.

Dr. Freeman moved that a committee be appointed to bring the subject before the next legislature.

Dr. Morgan favored the appointment of the committee, and gave his view as to how legislation can be best secured.

Dr. Chisholm suggested that the committee be instructed as to its duty. He favored a liberal law carefully drawn.

Dr. Cobb feels much interest, and thinks it a work hard to perform, favors the preparation of the proposed bill by the committee to come before this society, and then by memorial bring the matter before the legislature. Believes it to be a necessity.

Dr. Freeman thinks it will not be long before the law is secured. Thinks it ought to be done at once. Let the committee attend to the work and push the matter, and if we fail to do it at once, try again and again, until the work is done.

Dr. Morgan is in favor of making it a standing committee. Dr. Freeman amends his motion to a standing committee.

Dr. Claywell wants the matter agitated by and through the press, so the people may be prepared for it.

Dr. Cobb approves a memorial from the people. Thinks the people if they have the facts put before them will favor the move.

Dr. Shepard said the subject matter had been discussed among his friends, and they all favor it if a just and equitable law can be enacted.

Vote was put and carried.

A discussion ensued in which Drs. Morgan, Cobb and others made numerous suggestions.

Dr. Cobb moved that the committee be empowered to appoint as many dentists as is, in their opinion, necessary to co-operate with them. Carried.

Moved that the paper of Dr. Prothro be the order of the day at two o'clock. Carried.

Moved and carried that we hold an evening session at eight o'clock, and proceed immediately to the election of officers.

AFTERNOON SESSION.

Association called at half past two o'clock.

Reading of the minutes of the morning session, on motion, dispensed with.

Dr. Prothro read a paper on Diagnosis.

Dr. Chisholm endorsed every word in the paper, and felt thankful there are true men who have a lively sympathy for their patients.

Dr. Freeman endorses the paper, thinks the dangers referred to are imaginary. A systematic effort on our part to keep our patients cheerful will do much to make the operation bearable.

Discretion on the part of the dentist will always dictate to him when it is best to extract in pregnancy.

Dr. Russell is delighted with the paper. Thinks it has the right ring. Thinks gentlemanly dignified kind treatment to our patients in pregnancy, is the duty of every one dealing with such sensitive organs.

Dr. Noel endorses the paper. Persons who come to us in a state of suffering should of all others be most carefully and tenderly treated. We sometimes have to destroy tooth pulps of pregnant patients, and they are the hardest to overcome.

Dr. Morgan—I was taught that the time of pregnancy was one of great danger in operating. I think it is much exagerated. In thirty years practice I have never known a case of accident from operating for pregnant women, but while the danger is not imminent and bad results rarely occur, we should always be as careful as we can. I plead guilty when I think of my efforts to obtund sensitive dentine. Ether spray is the best obtunder in my hands. It is an exceedingly difficult thing to deal with. The diagnosis covers the whole field of physiology and pathology.

As a rule women in pregnancy should not submit to a dental operation unless it is a necessity. And it is the duty of our profession to inform them at what time operations may be made.

Dr. Ross did not hear the paper read, but from the discussion sees its bearing. Has never seen or known any bad results either from filling or extracting teeth for pregnent women in a long and constant practice.

Dr. Morgan—As a rule, if the indications are that the patient will have a long, continuous pain, I would extract at any stage of pregnency.

Dr. Prothro said several well authenticated cases of congenital deformity from dental operations had come under his observation.

Dr. Morgan referred to one case.

Dr. Chisholm has never known an accident from dental operations while pregnant. Any fright or painful influence about the time her catamenial periods would come on, if in health, is always the time most dangers and congenital deformities or marks are liable to occur. Stated several cases that had come under his observation. Said care and firmness was all that was necessary.

Dr. Freeman related one very serious case of abortion which happened in this city, in which the patient, three months gone, had miscarried while on the way from the office.

Dr. Morgan said the probability of abortion at regular periods is greater. It is more likely to occur then than at any other time.

Dr. Crawford asked if there was more or less danger of abortion in extraction of teeth under an anæsthetic.

Dr. Noel said he would, if desired, give ether.

Dr. Duval—This to me has been a very pleasant meeting, and the kindness and courtesy extended to young members of the profession is appreciated. The discussion of the paper of Dr. Prothro reflects credit on this body. It has impressed me forcibly.

Subject passed.

Subject of mechanical dentistry was next taken up, that of materials for filling teeth being passed.

Dr. Prewett said he was using rubber exclusively. Celluloid having been a failure in his hands.

Dr. Walker said his early experience with celluloid was not very pleasant. Then used rubber for sometime. Have found difficulty in making celluloid unite, but now succeeds very well by the use of spirits of camphor. Described his method in detail.

Dr. Beach said—I think celluloid fills its place in artificial dentistry. It is a most excellent thing for temporary teeth. It is so exceedingly temporary that sometimes it takes two or three temporary sets to last until the mouth is done shrinking. The best work is made on gold plate with rubber attachment.

Dr. H. Morgan said he liked Dr. Walker's idea of duplicating plates. Thinks celluloid is exceedingly temporary.

Dr. Crawford said his celluloid plates had a tendency to crawl around. Liked the idea of rubber attachment to gold plate.

Dr. Morgan said in articulation of teeth he made them so the front teeth do not come in contact with the lower, when the teeth come naturally together makes them so when they come together they do not move either way. One of the most serious troubles with artificial teeth, as I see them, is the circle is too large, the bicuspid teeth are made too prominent.

Dr. Chisholm—I want to add my testimony to what Dr. Beach said about gold plates. The Doctor said the majority of lower teeth will cut the gum in front because the front teeth cause the plate to move, which I endorse. Air chambers are of no use.

Dr. Russell said rubber or celluloid should always be moulded between metals. He likes gold with rubber attachments, thinks it the most beautiful work made. The greatest trouble is in getting the bite, and the best way is not to tell your patient of their difficulty of biting, but when ready say quickly bite, and it will be natural; told of some interesting cases.

Dr. Ross—Did I understand Dr. Chisholm to say he did not use air chambers. Dr. C., yes sir. Dr. Ross said he used thinner air chambers every year. Puts plumpers or a thick rim on the outside of plates.

Dr. Freeman—I think I have discovered the cause of shrinkage of rubber from the pins. When the heat reaches two hundred and sixty degrees turn your flame down and heat up slowly to two hundred and eighty degrees, then hold at that point from ten to fifteen minutes, then you may raise the heat to three hundred and sixty degrees if you like, and finish in fifteen minutes. This discussion was now closed.

EVENING SESSION.

The meeting commenced at eight o'clock. The order of business was the election of officers, which being held, the following were elected officers for the ensuing year, viz:

S. M. Prothro, President.

A. F. Claywell, First Vice President.

J. Y. Crawford, Second Vice President.

R. R. Freeman, Recording Secretary.

S. J. Cobb, Corresponding Secretaay.

W. H. Morgan, Treasurer.

L. G. Noel, A. S. Duval, J. C. Ross, Executive Committee.

R. R. Freeman, H. E. Beach, W. H. Morgan, Committee on Legislation.

On motion the treasurer was ordered to pay the bills incurred to defray the expenses of this meeting. A vote of thanks was given Col. Ed. S. Wheat, also to railroads and hotels for courtesy.

Adjourned to meet in June, 1879, in Nashville.

H. E. Beach,
Secretary.

